# Fatigue Induced by Alarm Sounds among Nurses Working in Intensive Care Units: A Descriptive and Analytical Study

**DOI:** 10.1192/j.eurpsy.2026.11226

**Published:** 2026-07-31

**Authors:** A. Feki, I. Sellami, A. Abbes, A. Haddar, M. L. Masmoudi, M. Hajjeji, K. J. Hammemi

**Affiliations:** 1 Rheumatology Department; 2 Occupationnel Medicine Department, Hedi Chaker Hospital, Sfax, Tunisia

## Abstract

**Introduction:**

In modern intensive care units (ICUs), nurses are continuously exposed to numerous auditory alarms emitted by monitoring and life-support equipment. Although designed to ensure patient safety, excessive alarm frequency and false alarms contribute to *alarm fatigue*—a progressive desensitisation that can impair vigilance, increase mental workload, and endanger patient outcomes. Alarm fatigue has emerged as a significant occupational health issue, often associated with psychological stress and physical exhaustion. Assessing the impact of alarm exposure on nurses’ fatigue levels is crucial for developing effective prevention and management strategies.

**Objectives:**

This study aimed to describe and analyse the level of fatigue induced by alarm sounds among ICU nurses and to explore its association with perceived stress. Specifically, it sought to quantify fatigue using the Fatigue Assessment Scale (FAS) and measure perceived stress through the four-item Perceived Stress Scale (PSS-4)

**Methods:**

A descriptive and analytical cross-sectional study was conducted among ICU nurses working in different hospital units. Participants were selected through convenience sampling. Data were collected using a self-administered questionnaire including sociodemographic variables (age, gender, years of experience, shift pattern) and the FAS, PSS-4. Statistical significance was set at p < 0.05

**Results:**

A total of 98 ICU nurses participated in the study, with a mean age of 34,69 years and a mean professional experience of 6.57 years. The male-to-female ratio was 1.2 (55/43). Our study revealed that a substantial proportion of nurses (85 participants, 86.7%) experienced fatigue. Most of them (74 participants, 75.5%) reported mild to moderate fatigue, whereas a minority (11 participants, 11.2%) exhibited severe fatigue. A low level of stress was observed in 76.5% of participants, with a mean perceived stress score of 6.46 ± 1.37. Bivariate analysis showed no significant gender difference in fatigue scores associated with noise exposure, nor any variation across hospital units. Nevertheless, alarm-related fatigue was found to be higher among nurses with longer professional experience

**Conclusions:**

This study highlights the substantial burden of fatigue among ICU nurses, particularly that induced by frequent and false alarms. Although gender and service type were not determining factors, professional seniority appeared to increase vulnerability to alarm-related fatigue. These findings emphasize the need for institutional strategies focused on alarm management, ergonomic optimization, and mental health support to preserve nurses’ alertness and well-being. Further longitudinal studies are recommended to explore causal mechanisms and evaluate targeted interventions.

**Disclosure of Interest:**

None Declared

